# Insights into soil bacterial and physicochemical properties of annual ryegrass-maize rotation (ARMR) system in southern China

**DOI:** 10.1038/s41598-021-99550-z

**Published:** 2021-10-11

**Authors:** Yanli Xiong, Xiaopeng Yang, Yi Xiong, Chaohui Xiong, Wenlong Gou, Xiao Ma

**Affiliations:** 1 College of Grassland Science and Technology, Sichuan Agricultural University, Chengdu, 611130 China; 2 College of Environmental Sciences, Sichuan Agricultural University, Chengdu, 611130 China; 3 Sichuan Academy of Grassland Science, Chengdu, 611731 Sichuan China

**Keywords:** Bacterial structural biology, Microbial communities

## Abstract

The popularized application of annual ryegrass—maize rotation (ARMR) in southern China has been proposed to fully utilize the farmlands and to increase forage yield and quality. Herein, one growth cycle of ARMR was conducted and soil bacteria were analyzed by 16S rRNA sequencing for control (CK), after the preceding crop (monoculture, or mixed sowing of annual ryegrass and oat) and the successive crop (maize). Our results indicated that the α-diversity of soil bacteria was changed in the ARMR system, which was related to the activity of urease and available phosphatase. The mixed sowing of annual ryegrass and oat in preceding crop could improve the yield and quality, while it was accompanied by unbalanced soil community. With the increased sowing proportion of oat to annual ryegrass, the soil pH increased while the soil available phosphatase decreased. The ARMR system was found to benefit the soil microenvironment by increasing the beneficial soil bacteria and enzyme activity or decreasing the harmful soil bacteria. Considering the soil bacteria α-diversity index and physicochemical properties comprehensively, the recommended sowing regime is the mixed sowing of M2 (22.5 kg·hm^−2^ annual ryegrass with 75 kg·hm^−2^ oat).

## Introduction

The interplay between crops, soil, soil microbial community and agricultural production practices is the principal limiting factor in agricultural production and sustainable development^1^. It is now universally accepted that soil microorganisms participate in a variety of soil biogeochemical processes, such as the decomposition of organic materials, formation of humic substances, nitrogen fixation, and improvement of soil aggregates^2^. The soil environment can provide nutrients such as minerals and organic matter for soil microorganisms, which in turn promote the biogeochemical cycling of carbon, nitrogen, and other elements through their metabolism. In addition, agricultural management practices such as the applied cropping system, nutrient input and tillage, have been shown to affect plant productivity and soil health by changing soil microbial communities and functions, as well as soil physicochemical modifications^3^.

Forage crop rotation is a management practice with great capacity to alleviate soil erosion, and to improve forage quality and yield^4^. Previous studies have shown that the main mechanism for forage rotation to increase yield is the complicated interaction between soil chemical and biological factors, such as the increased mineral nitrogen and available phosphorus, and a reduction in harmful microorganisms in the soil^5^. Theoretically, changes of the above-mentioned biochemical factors should be usually accompanied by changes in soil microbial diversity and community structure^6^. However, the variation of stubble type and length of the preceding crop will lead to the selective enrichment of soil bacteria for the successive crop, which makes the relationships between forage rotation and soil bacterial community structure complicated^7^. According to Alvey et al., in the rotation system, the primary bacteria related to the preceding crop will have a certain influence on the bacterial community of the successive crop^8^. Thus, the optimal choice of preceding crop and successive crop and their corresponding effects on soil microbial community dynamics are the determinants of high yield and sustainability in the rotation system.

The production system of annual ryegrass (*Lolium multiflorum* L.)—forage maize (*Zea mays* L.) rotation (ARMR) was implemented to make full use of the farmlands characterized by extensive traditional monoculture in southern China, which was assumed to have great production potential and economic benefits via efficient transformation by herbivores^9,10^. As a high-quality cold-season forage grass with high yield and good adaptability, annual ryegrass is subject to an expanding application in southern China due to its potential utility in rotation and intercropping with cereal crops. The production problems of annual ryegrass due to its weak growth vigor in the early stage and slow dry matter accumulation can be improved by mixed sowing with other fast-growing forages. Oat (*Avena sativa* L.) is a cereal crop of global importance that is used as grain, forage, and a rotational crop. Based on our previous findings (Table S1), the practice of mixed seeding of oat and annual ryegrass can fully utilize the existing natural resources, thereby improve the total biomass yield and quality in the early stage of growth. The current researches on the ARMR system have mainly focused on cultivation management measures and the effect of fertilizer synergist on N_2_O emissions during the process of rotation^11,12^. However, the dynamics of soil microbial diversity and community structure in the ARMR system has been rarely reported; even less the effect of oat to ryegrass ratio of the preceding crop on bacterial community and soil physicochemical properties.

The method of 16S rRNA sequencing of soil bacteria, which can annotate microbial species (genera) by blasting with the database, has the characteristics of wide application range, high sensitivity, and good dependability^13^. In this study, we applied one growth cycle of ARMR including control (CK), preceding crop and successive crop. Five treatments including monoculture of ryegrass (22.5 kg·hm^-2^, R0) and oat (150 kg·hm^-2^, O0), mixed sowing of annual ryegrass with oat of 37.5 kg·hm^-2^ (M1), 75 kg·hm^-2^ (M2) and 112.5 kg·hm^-2^ (M3) were applied in preceding crop. After the last sowing of preceding crop, 78,400 (D1) and 60,600 (D2) plants per hectare of maize were transplanted to each treatment of preceding crop. The soil physicochemical properties, enzyme activity and diversity and community structure soil bacteria of CK, after the preceding crop and successive crop were measured. This study aimed to (i) evaluate the dynamics of soil physicochemical properties, enzyme activity and bacterial communities on the ARMR system; (ii) detect the effect of mix ratio of oat to annual ryegrass on the soil bacterial community of successive crop; (iii) identify the relationships between soil physicochemical properties and the soil bacterial community.

## Results

### Soil bacteria α-diversity

**Figure 1:**
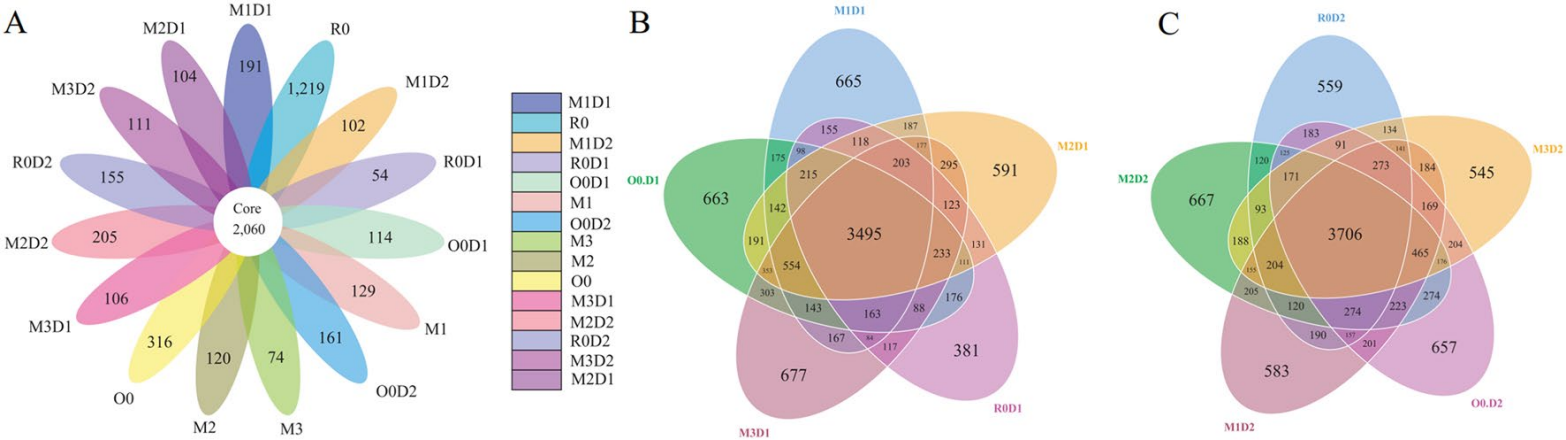
The Venn diagram of bacterial OTUs in the ARMR system. The shared and unique bacteria among all treatments (**A**), among the successive crop with D1 (**B**), and with D2 (**C**).

A total of 2,060 shared operational taxonomic units (OTUs) were identified among fifteen treatments in the ARMR (annual ryegrass and maize rotation) system (Fig. 1A, Table S2). The unique OTUs ranged from 54 (R0D1, with the monoculture of ryegrass in preceding crop and 78,400 plants per hectare of maize in successive crop, and so on) to 1,219 (one of two monoculture oat treatments, R0), and 3,495 and 3,706 OTUs were common among five treatments of D1 (78,400 plants per hectare of maize) and D2 (60,600 plants per hectare of maize), respectively (Fig. 1B,C).

**Figure 2:**
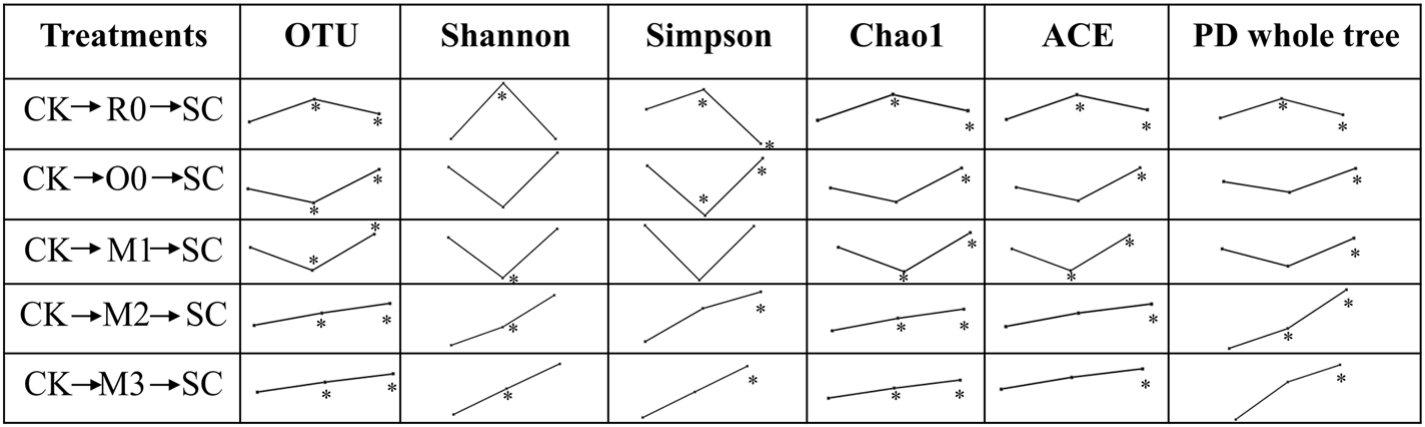
The change tendency in soil bacteria α-diversity indices in the ARMR system, with three replicates for each data. The same below. The asterisks indicated the significant differences of soil bacteria α-diversity indices between preceding crop/successive crop and CK with the level of *P* = 0.05.

Differences were found in the *α*-diversity indices of all soil bacteria among preceding crop, among which R0 demonstrated the highest value. A two-way ANOVA was used to evaluate the effects of planting density in the successive crop, type of preceding crop, and their interactions on the soil bacterial diversity indices of the ARMR system (Table S3). However, the preceding crop had a significant effect on Shannon index (*P* = 0.031). By comparing the soil microbial diversity indices of the CK, preceding crop and successive crop, it was found that the soil microbial diversity indices presented a trend of first increasing and then decreasing when the preceding crop was R0, while the soil microbial diversity indices presented an opposite trend when the preceding crop was O0 (one of two monoculture oat treatments) and M1 (annual ryegrass mixed sowing with oat of 37.5 kg·hm^−2^), i.e. first decreased and then increased. When the preceding crop was M2 (annual ryegrass mixed sowing with oat of 75 kg·hm^−2^) and M3 (annual ryegrass mixed sowing with oat of 112.5 kg·hm^−2^), however, the diversity indices of soil microorganisms showed a continuous upward tendency (Fig. 2). Remarkably, except Simpson diversity index in treatment with R0 in preceding crop, all the soil bacteria α diversity indices were increased after ARMR in all the treatments (Fig. 2).

### The relative abundance of soil bacteria

The ten phyla with the highest relative abundance for all treatments and the 35 genera with the highest total relative abundance were further analyzed (Fig. S1-A). The relative abundance of soil bacteria varied with the treatments. Specifically, eight soil bacteria genera (*Geobacter*, *Bacillus*, unidentified Clostridiales, *Alishewanella*, *Hyphomicrobium*, *Tahibacter*, *Truepera* and *Sphingopyxis*) were the most abundant in O0 (Fig. S1-A, Table S4), of which 62.5% (5 of 8) belonged to Proteobacteria. Compared to M2 and M3 treatments in successive crop, M1 possessed the higher abundance of *Methylobacterium*, *Acinetobacter* and *Rhodanobacter*, but the lower abundance of unidentified Acidobacteria. The abundance of unidentified Gammaproteobacteria increased, whereas six genera, including *Methylobacterium*, *Lactobacillus*, *Acinetobacter*, *Bacteroides*, *Stenotrophomonas* and *Rhodanobacter*, presented a decline in the successive crop of M1D1 and M1D2 compared to M1. The relative abundance of unidentified *Nitrospiraceae* elevated in M2D1 and M2D2 compared with M2, and that of unidentified *Nitrospiraceae* and *Terrimonas* increased in M3D1 and M3D2 compared with M3.

### Function prediction of soil bacteria in the ARMR system

The functions of bacteria in soil samples predicted by FARPROTAX analysis were mainly associated with the physiological and biochemical processes related to the carbon and nitrogen cycles and sulfide oxidation (Fig. S2-A). The main functions of soil bacteria in different planting techniques of the ARMR system (CK, preceding crop, successive crop·D1 and successive crop·D2) were variable. The soil bacteria of the CK were mainly shown to participate in fermentation, methylotrophy and ligninolysis (Fig. S2-B). The soil bacteria involved in the biochemical processes of nitrogen, sulfate, iron and manganese were diverse in R0, i.e., in one of two monoculture ryegrass treatments (Fig. S2-A), while the soil bacteria of O0 were mainly shown to participate in nitrate reduction and ureolysis. It is worth noting that the soil bacteria related to plant-pathogen interactions were abundant in M1, but showed low prevalence in other treatments. As the mixing proportion of oats increased in the preceding crop, the soil bacteria with functions of chemoheterotrophy and predatory or exoparasitic were decreased, but those participating in aerobic ammonia oxidation, aromatic compound degradation, and aerobic nitrite oxidation were significantly enhanced. As a whole, the differences in soil bacteria function between successive crop·D1 and successive crop·D2 were mainly in oxygenic photoautotrophy, hydrocarbon and aromatic compound degradation, etc. (Fig. S2-B).

### Soil bacterial community structure

**Figure 3:**
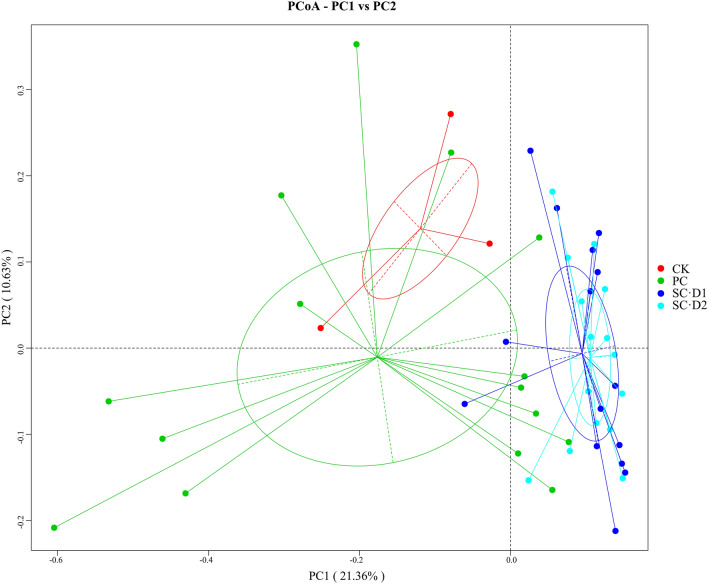
Soil bacterial community structure as described via PCoA analysis.

Principal co-ordinates analysis (PCoA) was applied to reveal the community structure of soil bacteria (Fig. 3). The first principal component (PC1) and the second principal component (PC2) totally explained 54.09% of variation, and also clustered together the SC·D1 and SC·D2 groups (Fig. 3). The analysis of similarities, multi-response permutation procedures analysis, and nonparametric multivariate analysis of variance all demonstrated a significant difference in bacterial community structure between D1 or D2 and CK or preceding crop (Table S5, *P* < 0.05). The difference between CK and preceding crop, or even D1 and D2 was not significant (*P* > 0.05).

**Figure 4:**
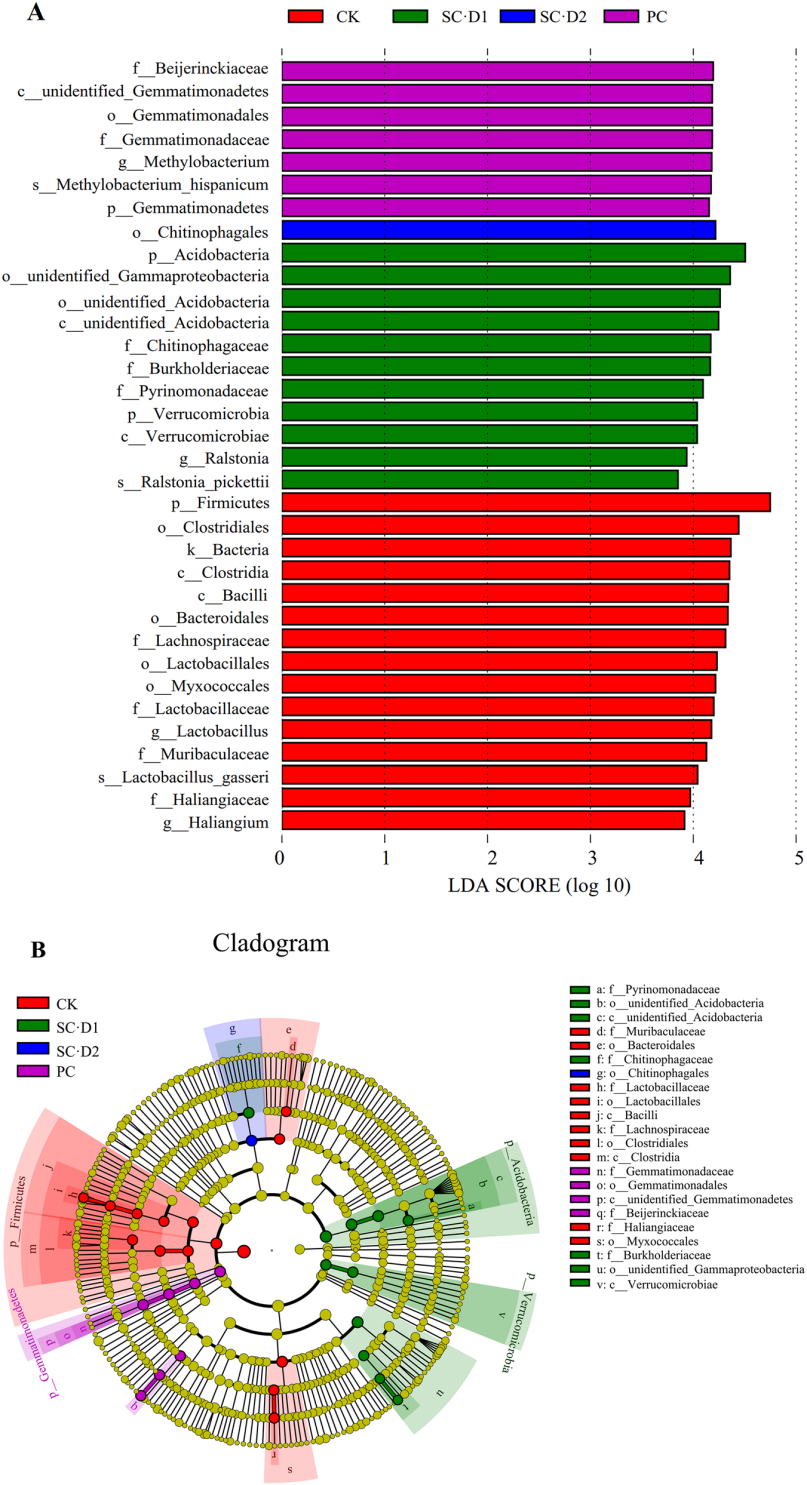
Line discriminant analysis effect size (LEfSe) between different treatments of the ARMR system. The circles radiating from the inside to the outside represent taxonomic levels from kingdom to species. Each small circle represents a taxon at that level, and the diameter of the small circle is proportional to the relative abundance.

Linear discriminant analysis (LDA) effect size (LEfSe) was applied to find biomarkers (taxa identified via the LEfSe analysis) (> 3.8 LDA score) with statistical differences between treatments (Fig. 4A). The result showed that CK possessed the maximum number of biomarkers, which belonged to Firmicutes, Bacteroidales, and Myxococcales (Fig. 4B). However, successive crop·D2 only featured the single biomarker Chitinophagales, and one of its members *Chitinophagaceae* was the biomarker of successive crop·D1. In addition to *Chitinophagaceae*, the biomarkers of successive crop·D1 consisted members of Acidobacteria, Verrucomicrobia and unidentified Gammaproteobacteria. Meanwhile, members of Gemmatimonadetes and *Beijerinckiaceae* were considered as the biomarkers of successive crop.

### Soil physicochemical properties and their correlation with bacterial α-diversity

**Figure 5:**
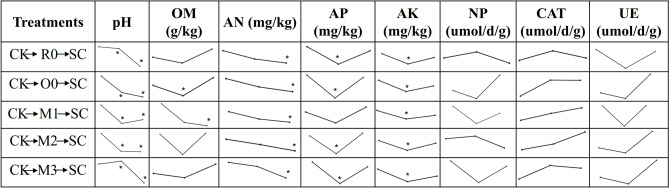
The change tendency in soil physicochemical properties in the ARMR system. The asterisks indicated the significant differences of soil bacteria α-diversity indices between preceding crop/successive crop and CK with the level of *P* = 0.05.

In general, CK soil possessed the highest values of organic matter, available nitrogen, available phosphorus, and available potassium in comparison to preceding crop (Table 1). The soil pH increased with the sowing proportion of mixed oats in the preceding crop and reached a peak value at O0 (pH = 7.54). The two-way ANOVA showed that the effect of maize planting density on all of the measured soil properties was not significant (*P* > 0.05). Only the effect of preceding crop type on the catalase activity of successive crop was shown as significant (*P* < 0.01). By comparing the soil physicochemical property indices of CK, preceding crop and successive crop, it was established that pH and available nitrogen had a continuous decreasing trend (Fig. 5). Except the organic matter content in treatment with M1 as the preceding crop, the soil physicochemical properties organic matter, available phosphorus, available potassium and urease activity showed a trend of first decreasing and then increasing. In short, catalase and urease activity were elevated after ARMR, while available nitrogen and available potassium were reduced. Although preceding crop tended to decrease the soil available phosphorus, available potassium and urease activity, the successive crop showed a compensatory effect.

**Table 1:** The soil physicochemical properties and enzyme activity in the ARMR system.

Treatment	pH	OM (g/kg)	AN (mg/kg)	AP (mg/kg)	AK (mg/kg)	NP (umol/d/g)	CAT (umol/d/g)	UE (umol/d/g)
CK	7.48 ± 0.12a	38.07 ± 0.81a	166.33 ± 1.33a	51.87 ± 2.45a	130.33 ± 1.20a	3.03 ± 0.42a	31.26 ± 2.03a	825.09 ± 14.77a
R0	7.44 ± 0.12a	28.93 ± 1.23ab	138.67 ± 10.17a	32.57 ± 8.22b	102.00 ± 6.36b	3.46 ± 0.02a	38.72 ± 5.17a	764.49 ± 29.75a
O0	7.54 ± 0.12a	26.17 ± 2.48b	140.33 ± 12.45a	22.70 ± 3.29b	100.00 ± 2.52b	2.91 ± 0.30a	46.72 ± 2.57a	813.43 ± 59.79a
M1	7.08 ± 0.05b	30.27 ± 1.92ab	141.00 ± 11.27a	37.17 ± 8.21ab	106.67 ± 14.26b	2.78 ± 0.53a	36.45 ± 9.61a	788.09 ± 10.63a
M2	7.20 ± 0.04ab	30.40 ± 1.08ab	150.00 ± 13.01a	24.97 ± 1.70b	102.67 ± 3.18b	3.22 ± 0.17a	37.84 ± 6.07a	816.64 ± 49.12a
M3	7.23 ± 0.15ab	30.43 ± 1.77ab	151.67 ± 5.46a	23.23 ± 1.27b	96.33 ± 4.06b	2.62 ± 0.18a	43.95 ± 3.61a	803.82 ± 37.17a
Mean	7.30	29.24	144.33	28.13	101.60	3.00	40.73	797.29
R0D1	7.00 ± 0.13	42.9 ± 4.99	133.07 ± 9.84	48.07 ± 7.05	127.33 ± 10.49	2.34 ± 0.63	32.96 ± 4.76	839.65 ± 43.40
O0D1	7.07 ± 0.05	36.4 ± 6.91	138.07 ± 13.46	47.33 ± 1.10	117.00 ± 3.06	3.70 ± 0.28	46.50 ± 2.17	915.69 ± 27.21
M1D1	7.24 ± 0.15	23.27 ± 1.42	130.37 ± 17.51	61.57 ± 16.75	118.33 ± 4.67	2.94 ± 0.37	45.32 ± 2.81	831.79 ± 52.41
M2D1	7.30 ± 0.07	29.23 ± 2.60	127.87 ± 11.36	61.67 ± 8.69	122.33 ± 10.40	2.28 ± 0.61	46.68 ± 1.55	844.6 ± 50.62
M3D1	7.10 ± 0.08	41.27 ± 3.09	102.30 ± 17.36	49.80 ± 3.71	109.33 ± 0.88	2.87 ± 0.63	38.83 ± 3.77	892.09 ± 22.97
Mean	7.14	34.61	126.33	53.69	118.867	2.83	42.06	864.76
R0D2	6.99 ± 0.06	35.47 ± 5.26	117.70 ± 14.29	46.60 ± 5.26	112.33 ± 6.74	2.98 ± 0.36	33.21 ± 1.26	796.53 ± 52.26
O0D2	7.25 ± 0.09	39.43 ± 8.98	113.17 ± 5.97	50.17 ± 8.76	113.00 ± 3.06	2.83 ± 0.36	46.49 ± 3.46	812.56 ± 65.85
M1D2	7.08 ± 0.16	31.97 ± 2.30	130.37 ± 5.93	55.10 ± 2.97	115.00 ± 5.03	2.92 ± 0.38	40.95 ± 0.93	820.42 ± 16.24
M2D2	7.07 ± 0.20	35.20 ± 5.22	134.87 ± 27.83	50.90 ± 11.99	123.33 ± 10.48	2.49 ± 0.25	48.35 ± 3.34	860.34 ± 26.35
M3D2	7.11 ± 0.07	38.20 ± 0.85	117.67 ± 1.20	42.07 ± 1.11	113.33 ± 4.06	2.99 ± 0.16	44.02 ± 0.88	870.82 ± 40.45
Mean	7.10	36.05	122.75	48.97	115.40	2.84	42.60	832.14
PD	F = 0.329	F = 0.704	F = 0.155	F = 0.821	F = 0.658	F = 0.003	F = 0.095	F = 1.469
	*P* = 0.573	*P* = 0.411	*P* = 0.698	*P* = 0.376	*P* = 0.427	*P* = 0.958	*P* = 0.761	*P* = 0.240
PC	F = 0.838	F = 2.367	F = 0.712	F = 0.923	F = 0.856	F = 1.162	F = 8.377	F = 0.765
	*P* = 0.517	*P* = 0.087	*P* = 0.593	*P* = 0.470	*P* = 0.507	*P* = 0.357	*P* = **0.001**	*P* = 0.560
PD × PC	F = 0.901	F = 0.939	F = 0.652	F = 0.214	F = 0.572	F = 0.816	F = 0.757	F = 0.552
	*P* = 0.482	*P* = 0.462	*P* = 0.632	*P* = 0.927	*P* = 0.686	*P* = 0.530	*P* = 0.565	*P* = 0.700

The data are shown as mean ± standard error (SE), with three replicates for each data. OM, organic matter content; AN, available nitrogen; AP, available phosphorus; AK, available potassium; NP, neutral phosphatase activity; CAT, catalase activity; UE, urease activity.

### The correlation between soil bacteria α-diversity indices and soil physicochemical properties

**Figure 6:**
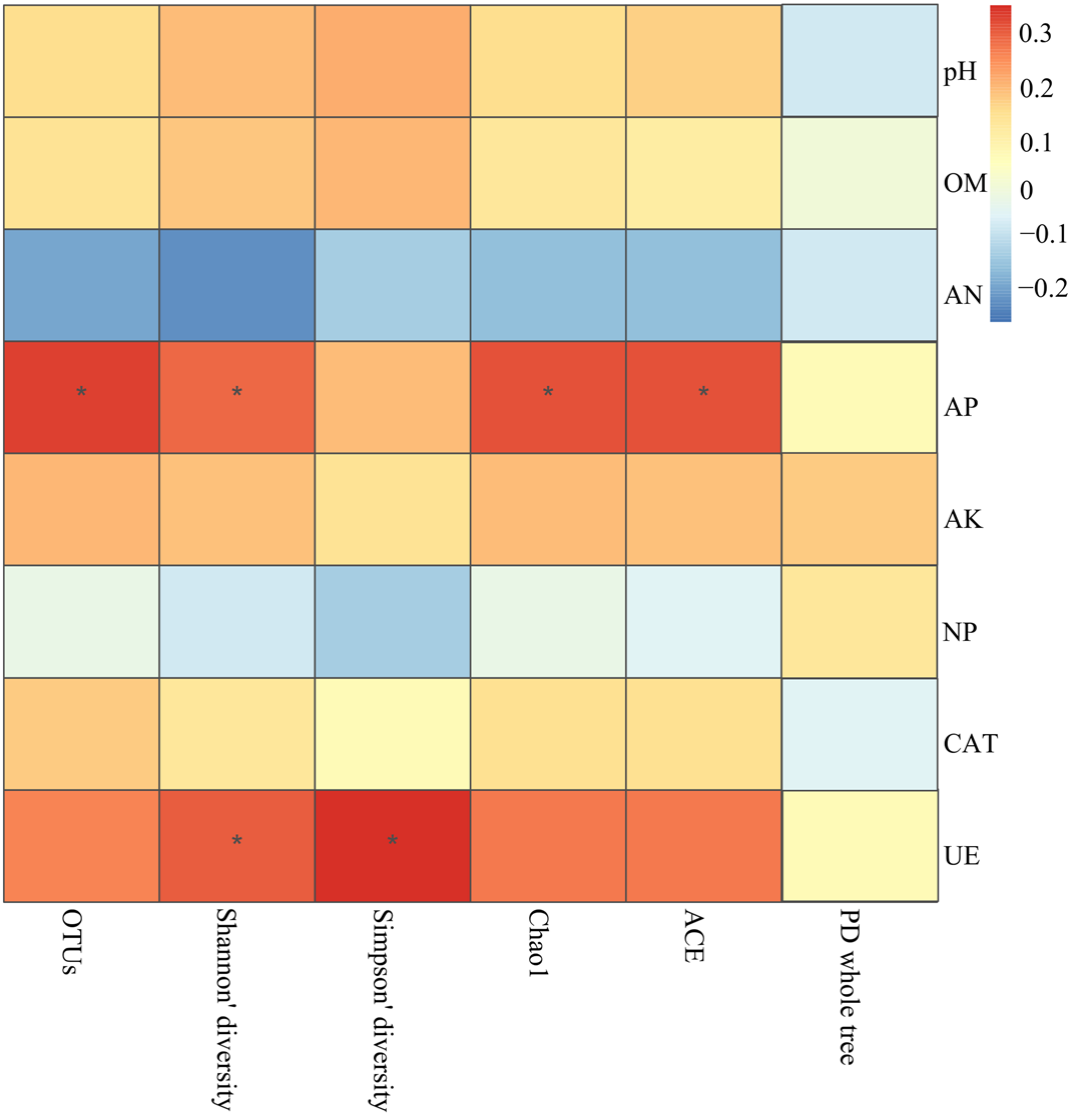
Spearman analysis showing the correlation between soil bacteria α-diversity indices, soil physicochemical properties, and enzyme activity in the ARMR system. The different color indicated the different correlation coefficients. The asterisks indicated the *p* values were less than 0.05.

The soil physicochemical properties showing the strongest correlation with the relative abundance of soil bacteria, screened via VIF and BioENV, were urease activity, and available nitrogen and available potassium content. According to Spearman correlation analysis, significant positive correlations were observed between available phosphorus content and OTUs, Shannon diversity, Chao1 and ACE (abundance-based coverage estimate) (*P* < 0.05, Fig. 6). The urease activity also significantly positively correlated with the Shannon and Simpson diversity indices (*P* < 0.05).

**Figure 7:**
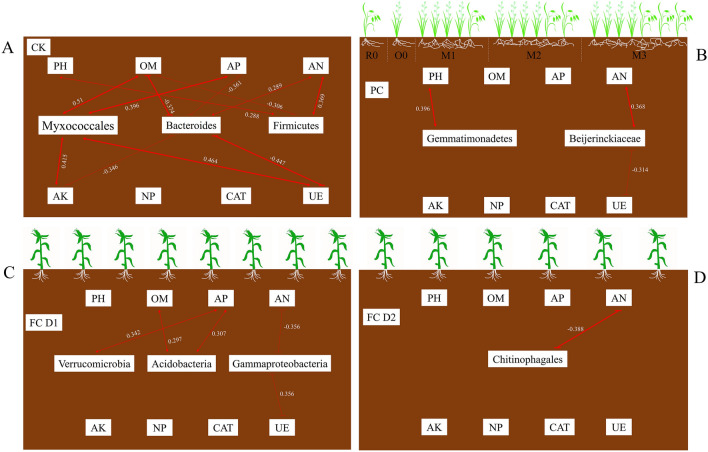
Spearman analysis showing the correlation between soil bacteria biomarkers and soil physicochemical properties or enzyme activity in CK (**A**), Preceding crop (**B**), SC·D1 (**C**), and SC·D2 (**D**). The numbers express the correlation coefficient. The red arrows indicate *p* values < 0.05 and the bold red arrows indicate *p* values < 0.01. PH: potential of hydrogen; OM, organic matter content; AN, available nitrogen; AP, available phosphorus; AK, available potassium; NP, neutral phosphatase activity; CAT, catalase activity; UE, urease activity. The lines end in arrows indicated the positive correction and lines end in horizontal line indicated the negative correction.

In order to look into the possible reasons for the formation of dominant bacteria at each planting stage (CK, preceding crop and successive crop), the associations between the relative abundance of biomarkers identified via LEfSe analysis and the soil physicochemical properties or enzyme activity were evaluated. The result showed that the relative abundance of Myxococcales in CK was significantly positively correlated with the UE activity and the content of organic matter, available potassium and available phosphorus (*P* < 0.01, Fig. 7). The Bacteroidales in CK, however, had a significantly negative correlation with urease activity, organic matter (*P* < 0.01), available potassium and available phosphorus (*P* < 0.05). The Firmicutes in CK showed a significantly positive correlation with pH (*P* < 0.05) and available nitrogen (*P* < 0.01) while its correlation with organic matter was significantly negative (*P* < 0.05). The members of Gemmatimonadetes and *Beijerinckiaceae* specific for preceding crop differed from those for CK and the successive crop, and were significantly positively correlated with the soil pH and available nitrogen, respectively (*P* < 0.01). For the successive crop·D1 treatment, the specific Acidobacteria had a significantly positive correlation with organic matter and available phosphorus (*P* < 0.05), the specific Gammaproteobacteria had a significantly negative correlation with available nitrogen and urease activity (*P* < 0.05), and the specific Verrucomicrobia had a significantly positive correlation with available phosphorus (*P* < 0.05). Furthermore, for the SC·D2 treatment, a significant correlation was only found between available nitrogen and the relative abundance of Chitinophagales (r = -0.338, *P* < 0.01).

The correlations among soil bacteria species, field samples (experimental treatments), and environmental factors (soil physicochemical properties and enzyme activity) were analyzed via Canonical Correlation Analysis (CCA) (Fig S3), which could explain 35.2% of variation in CCA1 and 18.87% of variation in CCA2. The most important environmental factor that affected samples and soil bacteria was catalase activity, followed by organic matter, available nitrogen, available phosphorus, available potassium, pH, neutral phosphatase activity and urease activity. Specifically, the samples M3, M3D1, M2, M2D1, M1D2, O0D2, O0D1, R0D1, M3D2, and soil bacteria *Deltaproteobacteria bacterium CSP1 − 8* were closed related to the soil catalase activity. The soil environmental factor neutral phosphatase activity mainly affected *Nitrospira japonica*. The field samples R0 and CK were primarily influenced by organic matter and available nitrogen, respectively, however, no other distinct relationships were found between other field treatments, soil bacteria, and soil environmental factors.

## Discussion

Soil bacteria constitute a major group of soil microorganisms, and their diversity and community structure determine the health and vitality of the soil^3^. However, the composition of soil bacterial community is complex in farmland systems and highly depends on the crop type, tillage, and management practices^14^. The soil bacteria α-diversity and community structure in the ARMR (annual ryegrass and maize rotation) system were studied in this study, which revealed the effects of forage rotation on the dynamics and ecological function of the soil bacterial community.

In the ARMR system of the present study, Proteobacteria had the highest abundance followed by Firmicutes, Bacteroidetes, Cyanobacteria, and Acidobacteria. This rotation system had an overall significant impact on the composition and community structure of soil bacteria. In general, the α-diversity of soil bacteria after rotation increased compared with that for CK (control, Table S3), which indicated the increased physiological and metabolic activity in the ARMR cropping system. According to Wang et al., mixed seeding results in higher soil bacterial diversity than monoculture^15^. However, in this study, the highest bacterial α-diversity in the preceding crop was observed in R0 (monoculture of annual ryegrass) as opposed to the mixed sowing treatments (M1, M2 and M3), which may be due to the high degree of overlap in the nitrogen preference of annual ryegrass and oat. Furthermore, the root distribution depth of both is concentrated on the soil layer of 0–20 cm, thus leading to obvious space and nutrient competition and even to the imbalance of soil bacterial community^16^. The present study detected the improvement of dry matter yield and forage quality (content of crude protein and water-soluble carbohydrates) in the early growth stage of annual ryegrass-oat mixed sowing system (Table S1). Nonetheless, most of the significant negative correlations were also detected between the above-mentioned yield and quality factors and the relative abundance of the 35 most abundant bacteria (Table S6), which further demonstrated that the mixed seeding of annual ryegrass and oat could improve the forage yield and quality, but was accompanied by low-level soil bacteria α-diversity. Notably, the soil pH increased and the soil available phosphorus decreased with the proportion of oat in the successive crop. According to Fan et al.^17^, oat has a preference for nitrate nitrogen thus enriched in an alkaline soil environment, which would reduce the utilizability of soil available phosphorus.

The PCoA analysis (Fig. 3) distinguished successive crop from CK and preceding crop, which indicated the effects of rotation on soil bacterial community structure. Changes in the bacterial community diversity and structure will affect their ecological function^18^. The abundance of the some Proteobacteria (Sphingomonas, Alishewanella, Acinetobacter and Sphingopyxis), which play a crucial role in the degradation of plant disease residues, promotion of nitrogen utilization, carbon and nitrogen fixation and cellulose degradation, etc., significantly increased after D2 treatment (*p* values were 0.013, 0, 0.003 and 0). However, a decreasing trend was observed in the soil bacteria Verrucosispora (Fig S1), which can produce exotoxins and thus cause certain plant diseases^19^. Therefore, ARMR is expected to benefit the microbial community system by promoting beneficial soil bacteria or by reducing harmful soil bacteria. This occurrence may be associated with the favorable interaction of the maize root and organic matter composition resulting from the decomposed stubble of the successive crop in this study.

Forage rotation influences soil bacteria α-diversity, which in turn affects soil properties such as enzymatic activity and element content^20^. In this study, the content of available potassium and available phosphorus, as well as the activity of urease and catalase of the successive crop increased distinctly compared to the preceding crop (Table 1), of which available phosphorus and urease were significantly associated with bacteria α-diversity indices (Fig. 5). The enhancement of available phosphorus and available potassium could increase the diversity and abundance of soil bacteria that are relevant to the function of adsorption and decomposition of phosphorus and potassium. On the contrary, changes in the microbial community may be a crucial mechanism affecting soil nutrient availability^21^. In addition, soil enzymes, especially urease and catalase, are important biological factors for the evaluation of soil substances, energy metabolism and soil quality^22^. Nitrogen produced from decomposition by urease is indispensable for the stability and improvement of bacterial diversity. The abundance of bacteria with nitrogen utilization function (nitrification and nitrogen fixation) was increased in this study, which further confirmed the correlation between urease and soil bacteria α-diversity. Therefore, we demonstrated that the ARMR system was beneficial to maintain the α-diversity of soil bacteria and soil enzyme activity, thus enhancing the stability of bacterial community and improving the ecological environment of the soil.

Fierer et al. and Li et al. reported that the content of organic matter was positively correlated with the abundance of Bacteroidetes, Gemmatimonadetes and Proteobacteria, but was negatively correlated with the abundance of Acidobacteria^23,24^. In this study, however, a significant positive correlation was established between organic matter and Myxococcales or Acidobacteria, and a negative correlation was observed between organic matter and Bacteroidales at the same time (Fig. 7). Given that the soil bacterial community is affected by complex factors such as vegetation type, climatic conditions, soil properties and human disturbance, our dissimilar results were not surprising. Previous studies have shown that members of the Gemmatimonadetes participate in sulfate reduction and low pH will reduce the abundance of Gemmatimonadetes^25,26^, which is in accordance with the highly significant positive correlation observed between the abundance of Gemmatimonadetes and soil pH in this study. The decreased soil pH during the progression of ARMR caused the enrichment of acidophil bacteria, such as Acidobacteria, Myxococcales and Verrucomicrobia, and the soil available phosphorus content increased with the reduction of pH at the same time, as mentioned above. Therefore, the significant correlation between available phosphorus, Acidobacteria, Myxococcales and Verrucomicrobia may be indirectly caused by the soil pH change^27^.

## Conclusions

The arable crop rotation providing soil with larger amounts of organic matter, e.g. through growing ryegrass-maize, exert many beneficial effects on soil properties and productivity. In summary, this work has shown that the soil bacteria α-diversity increased after rotation, which was positively related to the activity of soil available phosphorus and urease. The decreased abundance of harmful soil bacteria, as well as increased relative abundance of beneficial soil bacteria and enzyme activity were observed in the ARMR system, which demonstrates the agricultural sustainability of this farmland management practice in southern China via improving the ecological function of the bacterial community. However, a long-term and multi-site experiment is necessary for further investigating the response of soil bacterial communities to ARMR with the variation of soil type and climatic factors.

## Materials and methods

### Site description

One growth cycle of ARMR as a field experiment was carried out at Sichuan Academy of Grassland Science in Dayi County, Chengdu City, Sichuan Province, China (30°25′ N, 103°45′ E). The site has subtropical humid monsoon climate with annual average temperature and precipitation of 16.1℃ and 1,350 mm. The soil is yellow clay with pH of 7.48, organic matter content of 38.07 g·kg^−1^, Alkaline-nitrogen content of 166.33 mg·kg^−1^, available phosphorus content of 51.87 mg·kg^−1^, and Olsen-potassium of 130.33 mg·kg^−1^. The site had been fallowed for two years before the experiment was initiated.

### Experimental design

**Figure 8:**
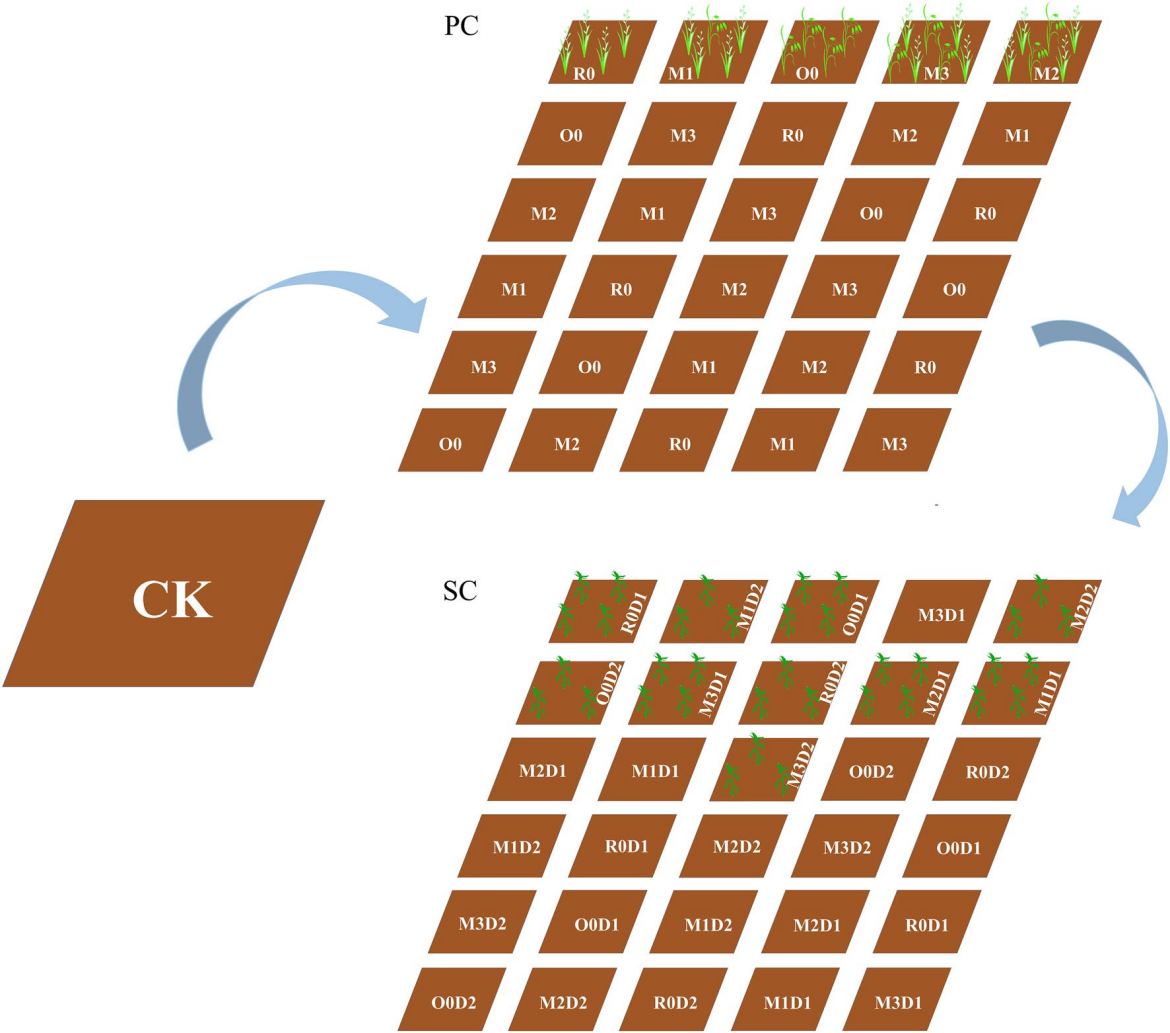
Diagram of experimental design of the ARMR system. CK, farmland fallowed for two years; PC, preceding crop, including O0 (22.5 kg·hm^−2^ ryegrass), R0 (150 kg·hm^−2^ oat), M1 (22.5 kg·hm^−2^ ryegrass mixed sowing with oat of 37.5 kg·hm^−2^), M2 (22.5 kg·hm^−2^ ryegrass mixed sowing with oat of 75 kg·hm^−2^) and M3 (22.5 kg·hm^−2^ ryegrass mixed sowing with oat of 112.5 kg·hm^−2^); SC, successive crop, including planting density of D1 (78,400 plants per hectare) and D2 (60,600 plants per hectare). R0D1 means treatment with R0 as preceding crop and D1 as successive crop, and so on.

The seeds of annual ryegrass (*Lolium multiflorum* L. cv ‘Tetragold’) and oat (*Avena sativa* L. cv ‘Qinghai No.444’) were both kindly provided by the Sichuan Province Grassland Work Station (Chengdu, China). The design of the field experiment is illustrated in Fig. 8. The preliminary experiment of mixed sowing of annual ryegrass and oat was carried out on 20 September 2018. This experiment had a completely randomized block design with six repeats. The area of each plot was 15 m^2^ (3 × 5 m). Five treatments including ‘Tetragold’ ryegrass seeded at 22.5 kg·hm^−2^ (R0), ‘Qinghai No.444’ oat seeded at 150 kg·hm^−2^ (O0), as well as annual ryegrass (22.5 kg·hm^−2^) in mixed sowing with oats of 37.5 kg·hm^−2^ (M1), 75 kg·hm^−2^ (M2) and 112.5 kg·hm^−2^ (M3) were performed. After the last mowing in 9 May 2019, the seedlings of maize (*Zea mays* L. cv ‘Yayu No.8’) were transplanted to each treatment of the preceding treatment with a sowing density of 78,400 plants per hectare (D1, 15 cm × 85 cm planting space) and 60,600 plants per hectare (D2, 30 cm × 55 cm planting space) with three repetitions. The density of D2 was based on the research of Zhang et al.^28^, and D1 was the recommended planting density of ‘Yayu No.8’. The 350 kg·hm^−2^ base fertilizer of N: P_2_O_5_: K_2_O = 15: 15: 15 was applied when transplanting, and 75 kg·hm^−2^, 265·hm^−2^ and 180·hm^−2^ of 46% N were applied at the 6–7-leave stage, big flare period, and heading stage, respectively.

### Soil sampling

The composite soil samples containing five soil cores of each plot were collected near the plants at 0–20 cm depth before the initiation of the preliminary experiment, at the end of the preliminary crop experiment, and at the harvest time of maize. The collected soil samples were immediately transferred into a portable refrigerator (Beijing, China). Each soil sample was then divided into two subsamples; one was air-dried and placed into a plastic bag after sieving through 1 mm screen for the determination of physical and chemical properties; the other was stored at −80 °C for DNA extraction and bacterial community analysis.

### Soil physical and chemical properties

A PHSJ 4A pH meter (Shanghai, China) was used for soil pH measurement with a soil to water ratio of 1: 2.5. Soil organic matter content was detected by the potassium dichromate method (K_2_Cr_2_O_7_). The alkali nitrogen-proliferation method, molybdenum-antimony anti-colorimetry, and flame photometry were applied for detecting the content of soil available nitrogen, available phosphorus and available potassium, respectively^29^. Micro Soil Urease Assay Kit (Solarbio, Beijing, China), Catalase Assay Kit (Solarbio) and Neutral Phosphatase Assay Kit (Solarbio) were used for the activity detection of soil urease, catalase and neutral phosphatase, respectively.

### 16S rRNA sequencing

The soil genomic DNA was extracted from 0.5 g soil subsamples using the DNeasy PowerSoil Kit according to the manufacturer’s protocol. The DNA purity was detected using 1% agarose gel, and the concentration was measured by a NanoDrop1 ND-1000 Spectrophotometer (USA). The DNA was diluted to 1 ng μL^−1^ for 16S rRNA gene sequencing. The PCR primer 515F/806R containing applicable adapters and 12-bp barcodes was used for Illumina sequencing of the V4-V5 region of 16S rRNA genes^30^. The PCR amplification was performed according to previously reported protocols with a total of 25 μL reaction volume. A dilution of 1 × loading buffer (containing SYB Green) mixed evenly with the same volume of the PCR amplification products was used for detection on 2% agarose gel.

Samples with a clear and bright main strip between 400 and 450 bp were selected for further testing, and these PCR products were purified by the Qiagen Gel Extraction Kit (Qiagen, Germany). The triplicate amplicon from each plot was pooled and sequenced on the Illumina HiSeq2500 platform with 2 × 250-bp pair-end sequencing. FLASH was used to merge the paired-end reads, and sequences containing ambiguous bases ‘N’ and over 90% with a sequencing quality < 30 were filtered^31^. High-quality clean tags obtained through the quality control process of QIIME^32^ were blasted to the Gold database using the UCHIME^33^ algorithm to detect the chimera sequences. The latter were removed, and the sequences with > 97% similarity were clustered into OTUs using UPARSE^34^. The sequence with the highest abundance in each OTU was selected as the representative sequence. Based on the GreenGene database^35^, the representative sequence was annotated using the RDP 3 algorithm^36^ to obtain the classified information of each OTU.

### Data analysis

The VennDiagram R package^37^ was employed to visualize the Venn diagram, which showed the common and particular OTUs of each sample. The ten most abundant bacteria taxa of each sample in the taxonomic groups of Phylum, Class, Order, Family and Genus were used to generate a histogram of the relative abundance of bacteria species. The α-diversity index (Shannon diversity), community richness [Chao1 index and ACE (abundance-based coverage estimate)] and phylogenetic diversity (PD whole tree) of soil bacteria were calculated using QIIME^32^, and visualized by the R package. Principal coordinate analysis (PCoA) with 999 permutations based on a Euclidean distance matrix was performed in the ade4 package of R^38^, which was used to uncover the dynamics of the soil bacterial community structure. Subsequently, the vegan R package was utilized for the analysis of similarities, non-parametric multivariate analysis (a new method for non-parametric multivariate analysis of variance) and multiple response permutation procedure. The LEfSe (linear discriminant analysis effect size) software was applied to discover the soil bacteria species whose abundance were significantly different among samples^39^. The functional annotation of prokaryotic taxa program was used to predict the function of soil bacteria^40^. The canonical correspondence analysis (CCA) with 999 Monte Carlo random permutations was carried out using the Canoco program^41^ to discover the environmental factors that best explained the functional gene composition. Normal distribution and homogeneity of variances were assessed using the Kolmogorov–Smirnov and Levene tests, respectively. One-way ANOVA followed by Tukey's post-hoc test was used to compare the difference of the soil physical and chemical properties.

### Ethical approval

All plants were kindly provided by the Sichuan Province Grassland Work Station (Chengdu, China).

### Informed consent

All local, national or international guidelines were adhered to in the production of this study.

## Supplementary Information

Supplementary Information 1.

Supplementary Information 2.

## Data Availability

The raw data of 16s rRNA sequencing was submitted in NCBI with accession number of PRJNA680198.
