# Supplementary material for: Insights into soil bacterial and physicochemical properties of annual ryegrass-maize rotation (ARMR) system in southern China
Source: Sci Rep. 2021 Oct 11;11:20125. doi: 10.1038/s41598-021-99550-z (PMC8505654; doi:10.1038/s41598-021-99550-z)
Supplement: Supplementary file 1 — Supplementary Information 1. [file 41598_2021_99550_MOESM1_ESM.pdf]

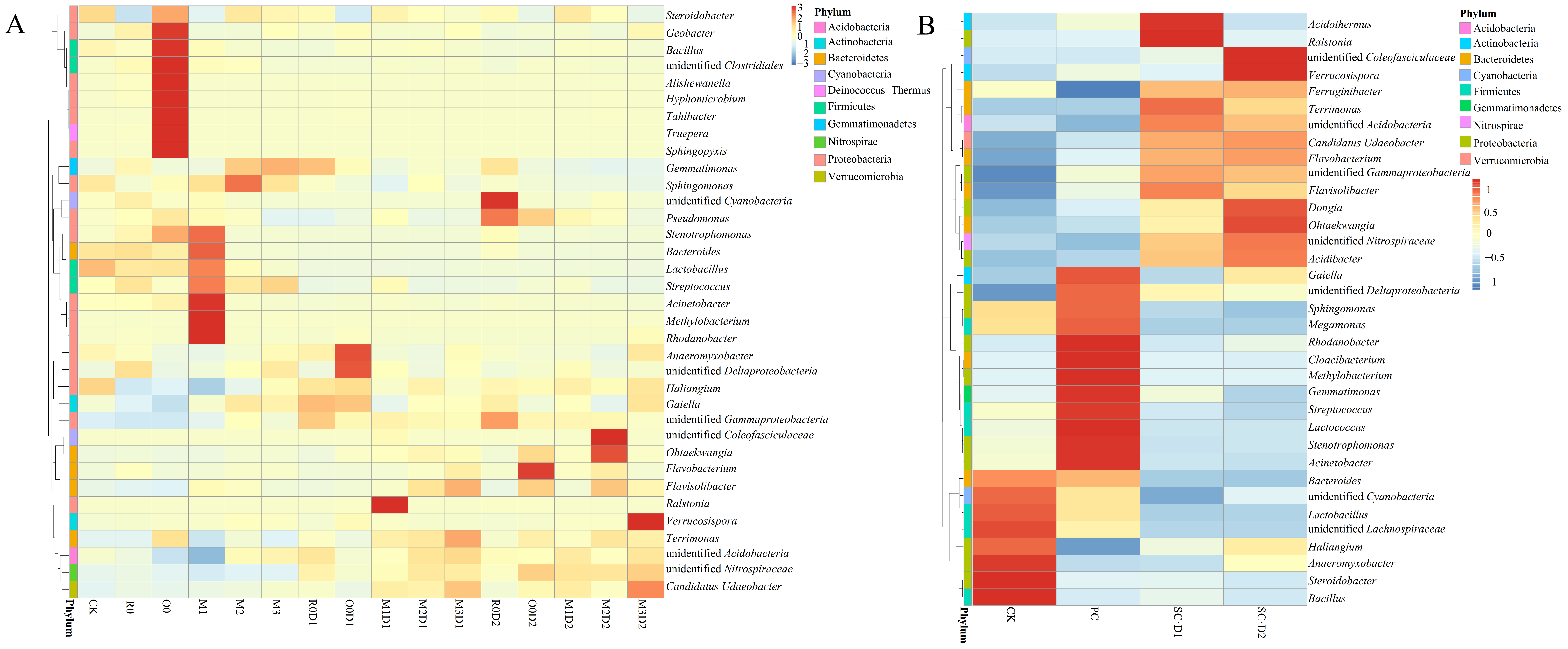

Fig S1 Heatmap showing the relative abundance of soil bacteria for each treatment (A) and for CK, PC, SC·D1 and SC·D2 (B).

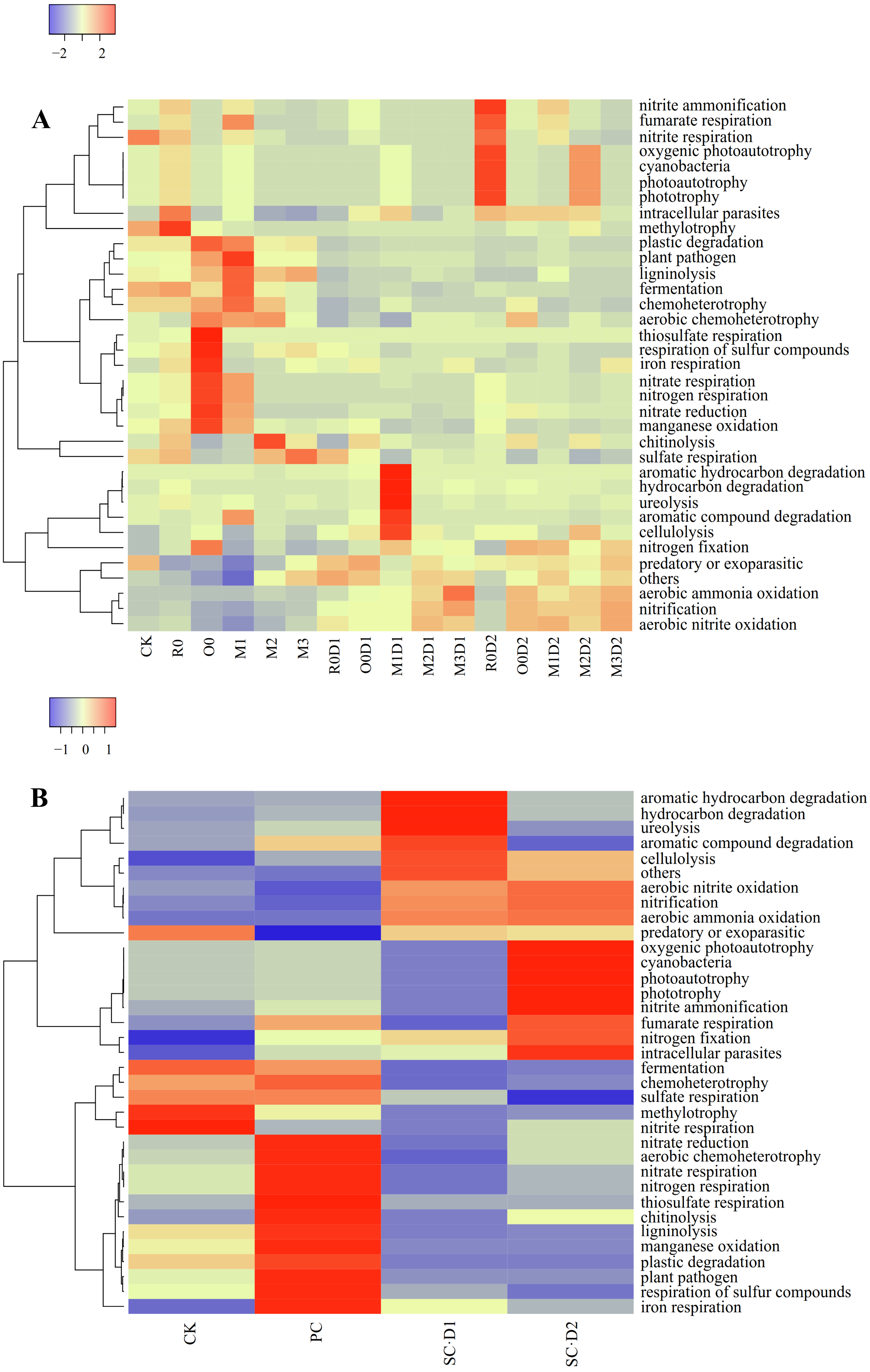

Fig S2: Heatmap of functional annotation of soil bacteria in the ARMR system.

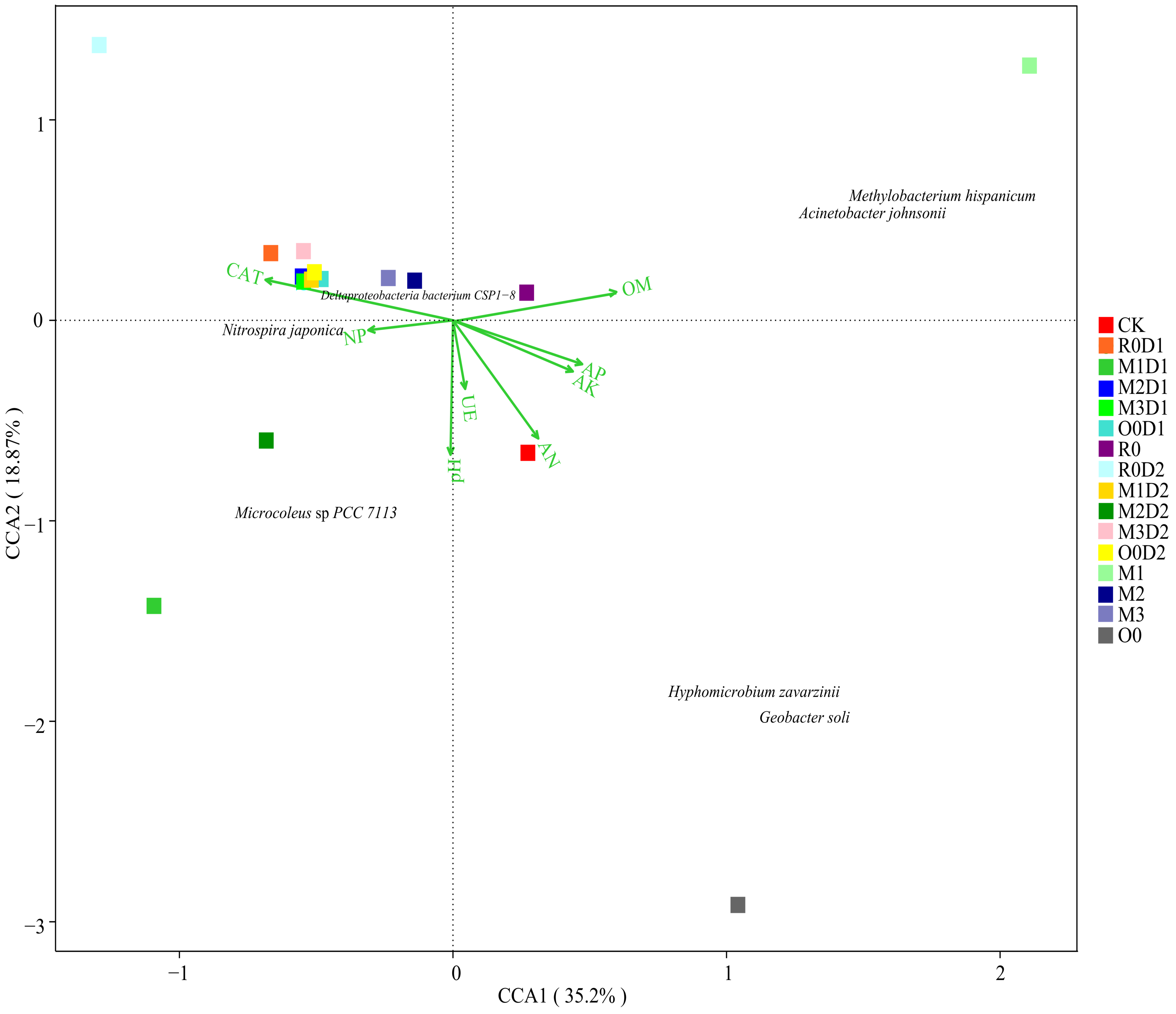

Fig S3: CCA plot showing the relationship among soil bacteria species, samples and environmental factors (soil physicochemical properties). The blue arrows indicate physicochemical properties and their length denotes the degree of association with soil bacteria and samples.
